# Ablation of pigment epithelium-derived factor receptor (PEDF-R/*Pnpla2*) causes photoreceptor degeneration

**DOI:** 10.1016/j.jlr.2023.100358

**Published:** 2023-03-17

**Authors:** Alexandra Bernardo-Colón, Lijin Dong, Mones Abu-Asab, Richard S. Brush, Martin-Paul Agbaga, S. Patricia Becerra

**Affiliations:** 1 Section of Protein Structure and Function, Laboratory of Retinal Cell and Molecular Biology, National Eye Institute, National Institutes of Health, Bethesda, MD, USA; 2 Genetic Engineering Core, National Eye Institute, National Institutes of Health, Bethesda, MD, USA; 3 Histopathology Core Facility, National Eye Institute, National Institutes of Health, Bethesda, MD, USA; 4 Department of Ophthalmology^,^ and Dean A. McGee Eye Institute, Oklahoma City, OK, USA; 5 Department of Cell Biology, University of Oklahoma Health Sciences Center, Oklahoma City, OK, USA

**Keywords:** Eye, eye/retina, PEDF-R/ATGL, phospholipase A2, phospholipids, photoreceptors, *Pnpla2*, retina

## Abstract

Photoreceptor cells express the patatin-like phospholipase domain-containing 2 (*PNPLA*2) gene that codes for pigment epithelium-derived factor receptor (PEDF-R) (also known as ATGL). PEDF-R exhibits phospholipase activity that mediates the neurotrophic action of its ligand PEDF. Because phospholipids are the most abundant lipid class in the retina, we investigated the role of PEDF-R in photoreceptors by generating CRISPR *Pnpla2* knock-out mouse lines in a retinal degeneration-free background. *Pnpla2*^*−/−*^ mice had undetectable retinal *Pnpla2* gene expression and PEDF-R protein levels as assayed by RT-PCR and immunofluorescence, respectively. The photoreceptors of mice deficient in PEDF-R had deformities as examined by histology and transmission electron microscopy. *Pnpla2* knockdown diminished the PLA_2_ enzymatic activity of PEDF-R in the retina. Lipidomic analyses revealed the accumulation of lysophosphatidyl choline-DHA and lysophosphatidyl ethanolamine-DHA in PEDF-R-deficient retinas, suggesting a possible causal link to photoreceptor dysfunction. Loss of PEDF-R decreased levels of rhodopsin, opsin, PKCα, and synaptophysin relative to controls. *Pnpla2*^*−/−*^ photoreceptors had surface-exposed phosphatidylserine, and their nuclei were TUNEL positive and condensed, revealing an apoptotic onset. Paralleling its structural defects, PEDF-R deficiency compromised photoreceptor function in vivo as indicated by the attenuation of photoreceptor a- and b-waves in *Pnpla2*^*−/−*^ and *Pnpla2*^*+/−*^ mice relative to controls as determined by electroretinography. In conclusion, ablation of PEDF-R in mice caused alteration in phospholipid composition associated with malformation and malperformance of photoreceptors. These findings identify PEDF-R as an important component for photoreceptor structure and function, highlighting its role in phospholipid metabolism for retinal survival and its consequences.

Photoreceptor degeneration is a major risk factor for blindness and its prevalence is growing worldwide (1, 2). The loss of rods and cones in the retina can lead to visual impairment or entire loss of vision and is a contributor to conditions such as macular degeneration and retinitis pigmentosa. Photoreceptors are a specialized type of neuroepithelial cells that absorb light and convert it into an electrical signal in the initial stages of phototransduction. They have a unique morphology with an elongated outer segment, which consists of hundreds of tightly stacked membrane disks that contain photopigment rhodopsin proteins surrounded by phospholipids necessary for phototransduction. While the photoreceptors contain a high content of phospholipids (3, 4, 5), the complex role of phospholipid metabolism in maintaining healthy photoreceptors has not been fully delineated.

Several lines of evidence suggest that vision can be preserved by interfering with photoreceptor cell death and that natural inhibitors of cell death can prevent retinal degeneration (6). One of them is pigment epithelium-derived factor (PEDF), a protein that acts in retinal survival (7, 8, 9). The retinal pigment epithelium secretes this interesting factor preferentially from its apical side into the interphotoreceptor matrix, where it acts on photoreceptor cells (10). The importance of PEDF in the development, maintenance, and function of the retina is evident in several animal models of inherited and light-induced photoreceptor degenerations (8). Our laboratory has demonstrated that PEDF prevents retinal cell death via interactions with a membrane-linked receptor protein termed pigment epithelium-derived factor receptor (PEDF-R) that exhibits phospholipase A2 (PLA_2_) activity (11, 12). Photoreceptor cells express the patatin-like phospholipase domain-containing 2 (*PNPLA2*) gene that codes and produces the PEDF-R protein detected in these cells (11, 13). Upon binding PEDF-R, PEDF stimulates its PLA_2_ activity to liberate fatty acids and lysophospholipids from phospholipids, and this activity is critical for the survival and nerve regeneration effects of PEDF in ocular cells (11, 12, 14, 15, 16). The photoreceptor cells are enriched in omega-3 fatty acids, such as docosahexaenoic acid (DHA), known for their neurotrophic and retinoprotective properties (3). While phospholipid metabolism is critical for the homeostasis of photoreceptors (3), it is currently unclear whether the PEDF-R phospholipase is a molecular link between phospholipids and photoreceptor survival. Deletion of the *Pnpla2* gene in mice may prove useful to understand the role of PEDF-R, and phospholipids, in the survival of photoreceptors to prevent blindness. It must be noted that the amino acid sequence of the mouse PEDF-R is identical to the mouse adipose triglyceride lipase (ATGL) and desnutrin, which have been reported extensively in tissues outside of the eye (11). Here, we refer to the gene and its protein as *Pnpla2* and PEDF-R, respectively.

To study the effects of the *Pnpla2* deficiency on the photoreceptor and neural retinal function, we used the CRISPR/CAS9 technology to generate a *Pnpla2* knockout (*Pnpla2*^*−/−*^) mouse model on a background that is free of known retinal degeneration mutations, such as *rd8.* We hypothesize that loss of PEDF-R function in the retina results in the deregulation of phospholipid metabolism to cause morphological and functional defects. To explore this proposition, we performed extensive characterization of photoreceptor structure and function in the *Pnpla2* knockout mice deficient in PEDF-R using molecular biology, biochemical, morphological, and physiological evaluations. We discuss how PEDF-R, the PEDF/PEDF-R axis, and phospholipids can participate in maintaining healthy photoreceptor cells.

## Materials and Methods

### Animals

*CRISPR Pnpla2* (C57BL/6J) mice were generated by the Genetic Engineering Core at the National Eye Institute. The mice were confirmed to be *rd8*-free and used to generate the mutated line. All mice (2.5–7 months of age, mixed sexes) were maintained in the animal facility of the National Institutes of Health. All the experimental procedures were approved by the National Eye Institute Animal Care and Use Committee and were performed as per guidelines of the Association for Research in Vision and Ophthalmology statement for the Use of Animals in Ophthalmic and Vision Research, USA, and in accordance with the ARRIVE guidelines. All the experimental animals were maintained on a normal chow diet and a 12 h light/12 h dark cycle.

### Generating *Pnpla2* knockout by CRISPR-mediated genomic deletion in C57bl6/J background

A null mutant allele of the mouse *Pnpla2* gene was created by deletion of a 2.483 kb genomic fragment spanning from exons 2 to 8 and a part of exon 9, the last coding exon of the *Pnpla2* gene, with the CRISPR/Cas9 technology using a pair of guide RNAs (gRNA, for SpCas9, PAM=NGG) flanking the deletion region in the zygotes of C57BL6/J strain free of the *rd8* mutation. gRNAs were selected based on their positions nearing the target sites and ranking by the online gRNA selection tool (www.CRISPRscan.org) and synthesized with T7 in vitro transcription as described (17) and further tested for their efficiencies of in vitro cleavage and in-cell culture indel mutagenesis activities. For the in vitro cleavage assay, the genomic PCR product containing the target sites of selected gRNAs was incubated with SpCas9 protein (NEB, New England Biolabs, Ipswich, MA) by following manufacturer’s suggested protocol and analyzed on 2% agarose gel electrophoresis and stained with ethidium bromide (not shown). gRNAs were further tested for their efficiencies inducing indels at target sites in an immortalized mouse embryonic fibroblast (MEF) cell line engineered to carry a tet-inducible Cas9 expression cassette (Lijin Dong, unpublished). Upon confirmation of efficient target cleavage activity in MEF cells, two selected gRNAs were mixed with SpCas9 protein (PNA Bio, Thousand Oaks, CA) and microinjected into mouse zygotes as described (18). The two gRNAs used to generate the deletion allele were the upstream guide 5′GGGAGAGCAGGGCCGGGATC3′ and the downstream guide 5′CCCACTAAGAGGAGCCCC3′, respectively. Mice identified as F0 founders carrying the deletion mutation were further confirmed by Sanger-sequencing of the PCR product spanning the deletion area and were backcrossed to C57BL/6J mice for germline transmission of the deletion mutation. For identification of *Pnpla2* deletion allele, genomic DNA was isolated from tail clips and PCR reactions were performed using My Taq™ Extract-PCR kit (Bioline, Meridian Bioscience, catalog number BIO-2116). The PCR genotyping assay was performed with a common upstream primer 5′-GCCAAGTAGGTGATGGTTGAAGTAG-3′ in combination with the allele-specific downstream primers reverse-WT 5′CCAGGCGTCCATTGGCGCGCTC3′ for detection of the WT allele (526 bp), and reverse-CRISPR-deletion 5′-GGTAACATGCAGAAGTGAGGAAGG-3′ for the deletion allele (733 bp). F1 germline founders of the *Pnpla2-*KO deletion allele were also genotyped for the *rd8* allele by an allelic discrimination assay using qPCR as described (19). Genotyping for colony expansion was performed by Transnetyx, Inc.

### RNA extraction, cDNA synthesis, and quantitative RT-PCR

Gene expression analyses were performed with semiquantitative real-time PCR, as described before (20). Total RNA was purified from the mouse retina, RPE, and liver using the RNeasy® Mini (Qiagen, Inc., catalog number 74104) following the manufacturer’s instructions. Between 10-50 ng of total RNA were used for reverse transcription using the SuperScript III first-strand synthesis system (Thermo Fisher Scientific, catalog number18080051) following manufacturer’s instructions. Murine *Pnpla*2 mRNA levels relative to *Hprt* transcript levels were measured by the QuantStudio 7 Flex Real-Time PCR System using Taqman® gene expression assays and using the *Pnpla2* and *Hprt* oligonucleotides shown in the table above. For *Rho* (rhodopsin) and *Opn1mw* (opsin 1), mRNA levels were normalized to *18S* levels by quantitative RT-PCR using the QuantiTect SYBR Green PCR kit (Qiagen catalog number 204143). The sequences of the primers are in Table 1.

**Table 1:** Oligonucleotide sequences of primers used for qRT-PCR

*Gene name* (Protein)	Accession #	Forward Primer	Reverse Primer
*Pnpla2* (PEDF-R)	NM_001163689	5′-AGCTCATCCAGGCCAATGTCT-3’^a^	5′-TGTCTGAAATGCCACCATCCA-3’^a^
*Hprt* (hypoxanthine guanine phosphoribosyl transferase)	NM_013556	5′-ACTGTAATGACCAGTCAACAGGGG-3’^b^	5′-TGTATCCAACACTTCGAGGAGTCC-3’^b^
*Rps18* (ribosomal protein S18)	NR_003278.3	5′-GGTTGATCCTGCCAGTAG-3′	5′-GCGACCAAAGGAACCATAAC-3′
*Rho* (rhodopsin)	NM_145383.2	5′-TCACCACCACCCTCTACACA-3′	5′-TGATCCAGGTGAAGACCACA-3′
*Opn1mw* (opsin 1)	NM_008106.2	5′-GTACCACCTCACCAGCACCT-3′	5′-GGGTGTCCCAGAACGAAGTA-3′

a from Thermo Fisher Scientific catalog number Mm00503040_m1.

b from Thermo Fisher Scientific catalog number Mm00446968_m1.

### PLA2 activity assay

Phospholipase A_2_ (PLA_2_) enzyme activity in mouse retinas was determined by real-time fluorometric monitoring with BODIPY® PC-A2, a selective substrate for PLA_2_, using the EnzChek™ Phospholipase A2 Assay Kit (Invitrogen, catalog number E10217) and following the manufacturer’s instructions. Dissected mouse retinas were incubated in EnzCheck PLA2 reaction buffer at 110 μl per retina for 10 min at 4°C. The retina tissues were disrupted using a FisherBrand™ Model 50 Sonic Dismembrator (Fisher Scientific) set at 30 (Amplitude dial) for 15 s at 4°C, followed by centrifugation at 20,800 *x g* for 10 min at 4°C to remove particulate material. Freshly prepared clarified retinal samples were mixed at 50 μl per PLA_2_ reaction assay with 50 μl of freshly prepared substrate liposomes and incubated for 10 min at room temperature. The PLA_2_ activity was assessed by ratiometric detection of the changes in the emission intensity ratio at 515/575 nm with excitation at ∼460 nm using SpectraMax® iD5 (Molecular Devices). Bee venom PLA_2_ was used as a standard. Preliminary assays were performed to determine the optimum extraction buffer of the enzyme from the retinas for the reaction assay. The use of buffers reported previously for the enzyme (Tris-HCl- and phosphate-based) (11, 21) did not yield measurable activity, likely because they were not optimal for the substrate liposomes. Because the suspension of retinal extracts in PLA_2_ reaction buffer yielded measurable PLA2 activity, it was selected for this assay. The PLA_2_ activity ratios were plotted using Microsoft Excel software.

### Transmission electron microscopy

Transmission electron microscopy was performed, as described before (22). Briefly, mouse eyes were enucleated and doubly fixed in PBS-buffered glutaraldehyde (2.5% at pH 7.4) and PBS-buffered osmium tetroxide (0.5%) and embedded in epoxy resin. Sections (90 nm thick) were generated and assembled on 200-mesh copper grids, dried for 24 h, and double-stained with uranyl acetate and lead citrate. Sections were photographed with a JEOL JM-1010 electron microscope.

### Retinal phospholipids profiling

Retinas dissected from the same animal were pooled and used for total phospholipid analyses as previously described (23, 24). Briefly, retinal tissue was homogenized in 40% aqueous methanol and then diluted to a concentration of 1:40 with 2-propanol/methanol/chloroform (4:2:1 v/v/vol) containing 20 mM ammonium formate and 1.0 μM phosphatidylcholine (PC) (14:0/14:0), 1.0 μM phosphatidyl ethanolamine (PE) (14:0/14:0), and 0.33 μM PS (14:0/14:0) as internal standards. Samples were injected into a triple-quadrupole mass spectrometer (TSQ Ultra, Thermo Scientific) by using a chip-based nano-ESI source (Advion NanoMate) operating in infusion mode. PC lipids were measured using precursor ion scanning of m/z 184, PE lipids were measured using neutral loss scanning of m/z 141, and PS lipids were measured using neutral loss scanning of m/z 185. All species detected for each group are represented as a relative percentage of the sum based on their response values. Abundances of lipid molecular species were calculated using the Lipid Mass Spectrum Analysis software (University of Helsinki, Helsinki, Finland).

### Paraffin embedding and immunofluorescence

The left eyes from mice were enucleated and an incision was made in the cornea. The eyes were fixed in 4% Paraformaldehyde Aqueous Solution (Electron Microscopy Sciences, catalog number 15710) for at least 48 h, then subjected to dehydration and paraffin embedding (performed by the NEI Histology Core). Ten-micrometer sections were cut from paraffin-embedded tissues and used for Immunofluorescence. Slides containing retinal sections were deparaffinized by immersing the slides in various ethanol dilutions, then rinsed with PBS (pH 7.4, Quality Biological, Catalog number 119-069-101), followed by incubation in blocking buffer containing 0.5% normal donkey serum (Abcam, Catalog number ab7475), 0.5% bovine serum albumin (GoldBio, catalog number A-420-100), 0.1% Triton X-100 (Sigma, Catalog number T8787-100) in PBS pH 7.4 for 2 h at room temperature. The slides were incubated in the above solution, containing the primary antibody (diluted as in the table above) at 4°C for 16 h, then rinsed with PBS followed by incubations with their corresponding secondary antibody (dilutions as in the table above) for 1 h at room temperature. Antibodies used in the study are in Table 2. The slides were rinsed in PBS and mounted in Vectashield Mounting medium containing 1.5 μg/ml DAPI (4′,6-diamidino-2-phenylindole, Vector Laboratories catalog number H-2000). Retinal section images were acquired using an Olympus FV-1000 confocal microscope. All images were collected from different mice with identical magnification, gain and exposure settings, and the same retinal region. Fluorescence intensity was quantified using ImageJ as previously described (25). A rectangle was selected around regions of interest (ROIs), channels were split for multiple antibodies, threshold was adjusted, noise was de-speckled, and fluorescence intensity was measured. Each experimental group included three eyes.

**Table 2:** Antibodies used in the study

Antibody	Type & host	Application	Dilution	Company, Catalog Number
Anti-Rhodopsin	Monoclonal mouse	IF	1:100	Sigma, MAB5356
Anti-ATGL	Polyclonal rabbit	IF	1:1000	Proteintech, 55190-4-AP
Anti-Opsin blue	Polyclonal rabbit	IF	1:200	Sigma, AB-5407
Anti-Synaptophysin	Monoclonal rabbit	IF	1:250	Abcam, Ab52636
PKC alpha	Monoclonal mouse	IF	1:50	Novus Biological, NB600-201SS
Alexa Fluor 488	Goat anti-Mouse IgG (H+L)	IF	1:200	Thermo Fisher Scientific, A-11001
Alexa Fluor 555	Goat anti-Rabbit IgG (H+L)	IF	1:200	Thermo Fisher Scientific, A-21428

### Cell surface exposed phosphatidyl serine detection in vivo

To prepare PSVue-550 as a fluorescent probe for eyedrop administration, bis (zinc^2+^-dipicolylamine)-550, (PSVue®-550) from Molecular Targeting Technologies Inc (catalog number P-1005) was reconstituted in HBSS (Quality Biological, catalog number 114-062-101) according to the manufacturer’s instructions as previously described (26). The resulting 1 mM solution of PSVue®-550 was stored in the dark at 4°C and used within 14 days directly as an eyedrop. After 3–4 h of light onset the mice received eyedrops of 5–10 μl of either 1 mM PSVue®-550 in HBSS in the left eye or HBSS in the right eye as a control. The eyedrop volume was chosen such that the eye cavity was filled without spillage outside the eye, and it may need to be modified depending on eye size. To obtain *In Vivo* retinal images of fundi, mice were anesthetized and eyes were dilated with 1% tropicamide for 5 min and kept hydrated with GenTeal. Fluorescence was imaged on a Micron III retinal imaging microscope (Phoenix Research Labs, Pleasanton, CA) using an FF02- 475/50 nm excitation filter (Semrock, Inc. Rochester, NY). Using ImageJ, the average intensity of the fluorescence on the back of the retina was quantified.

### TUNEL assay

To detect photoreceptor cell death in retina paraffin sections, the Click-it Plus TUNEL assay detection kit (Invitrogen, Thermo Fisher Scientific, catalog number C10617) was used as previously described (27). Staining was carried out according to the manufacturer’s instructions. Confocal microscopy was performed on an Olympus FV-1000 confocal microscope.

### Electroretinography

Electroretinograms (ERG) were recorded in WT, heterozygous, and homozygous *Pnpla*2 mice using an Espion E2 system with ColorDome (Diagnosys LLC, Lowell, MA, USA) with a heated surface. Mice were dark-adapted overnight as previously described (13). Briefly, pupils were dilated with 1% tropicamide (Akorn, catalog number NDC: 17,478-101-12) for 10 min and mice were anesthetized with IP injection of Zetamine™ (Ketamine hydrochloride injection, USP; VetOne, catalog number NDC 13985-584-10) at 92.5 mg/kg, and AnaSed® (xylazine injection, Akorn Pharmaceuticals, catalog number NDC 59399-110-20) at 5.5 mg/kg, according to previously published methodology (13). Mice were placed on the heated surface and electrodes were placed in the mouth and a subdermal platinum needle electrode was placed in the back of the mouse to serve as a ground. Gold electrodes were placed on lubricated corneas. GenTeal eye gel with 0.3% Hypomellose (Alcon, catalog number NDC 0078-0429-57) was applied throughout the procedure to prevent corneal drying. Mice were then exposed to 15 flashes of 1 Hz, 1 cd-seconds per meter squared (cd.s/m^2^). Amplitudes for a-wave were measured from the stimulus to the trough of the a-wave, and b-wave amplitudes were measured from a-wave to b-wave trough or peak. The values from the a and b waves for both eyes were exported to Microsoft Excel, and both eyes were averaged to obtain amplitude values for each mouse. Each eye was separately exposed to 15 flashes.

### Optical coherence tomography retinal imaging

To image cross-sections of the retina in vivo, mice were anesthetized and pupils were dilated with 1% tropicamide for 5 min and GenTeal eye gel with was applied freely throughout the procedure to prevent corneal dryness. A contact lens was not used during image acquisition. Mice were placed on the rodent alignment stage. Optical coherence tomography (OCT) images, centered on the optic nerve head, were obtained from every eye. OCT volume scans were acquired in the automatic real-time mode, averaging 9 frames per image. Each volume covered 30 × 30 and consisted of 31 horizontal and 6 radial B-scans (768 A-scans each), 200 μm apart. Retinal thickness was measured manually from each OCT scan using Image J system software and averaged from each mouse.

### Experimental design and statistical analysis

Data were analyzed using GraphPad Prism version 9.4.1 for Windows (GraphPad Software, San Diego, California). All experimental groups were compared to each other using a two-tailed unpaired Student *t* test or one-way ANOVA using Dunette’s multiple comparison as we compared the mean of the control group with the other groups. All groups are shown (mean ± SEM). *P* values lower than 0.05 were considered statistically significant.

## Results

### PEDF-R levels in CRISPR-derived *Pnpla2*-KO mouse retinas

**Fig. 1:**
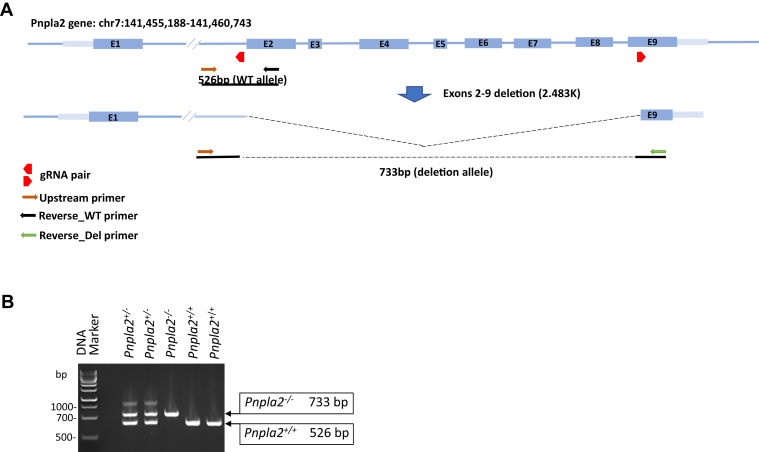
Generation of CRISPR-derived *Pnpla2*-KO. A: The *Pnpla2*-KO allele was generated by the deletion of a genomic region from upstream of exon 2 to mid-exon 9 removing most of the coding sequence of the target gene. The two guiding RNAs and genotyping primers used are as indicated. B: Gel electrophoresis of PCR-genotyping products obtained using the common upstream primer (brown arrow) and downstream primers for the WT allele (black arrow) and the KO allele (green arrow), respectively. Genomic DNA was isolated from tail biopsies of litter mates produced from heterozygous parents. The sizes of the amplicons obtained by PCR are indicated to the right of the photo of the gel. The genotypes are indicated at the top of each lane: *Pnpla2*^*+/+*^, WT; *Pnpla2*^*+/−*^, heterozygotes; and *Pnpla2*^*−/−*^, homozygotes. DNA size markers are AmpliSize Molecular Ruler (BIO-RAD).

The *Pnpla2*-KO allele generated through CRISPR-mediated genomic deletion in the C57BL6/J zygotes was germline transmitted as demonstrated by genotyping (Fig. 1). Given that the *rd8* mutation of the *Crb1* gene is present in several vendor lines of mice and embryonic stem cells, and its presence may confound ocular-induced mutant phenotypes (28), we also genotyped the F1 germline founders for the *rd8* allele and confirmed that the *rd8* mutation is not present (data not shown). We noticed that the *Pnpla2*^*−/−*^ mice did not survive past 3–4 months of age, like another reported model, *Atgl*^*−/−*^ mouse (29). Therefore, our next assessments were performed with homozygous mice at 2.5–3 months old and heterozygous mice at 7 months of age.

**Fig. 2:**
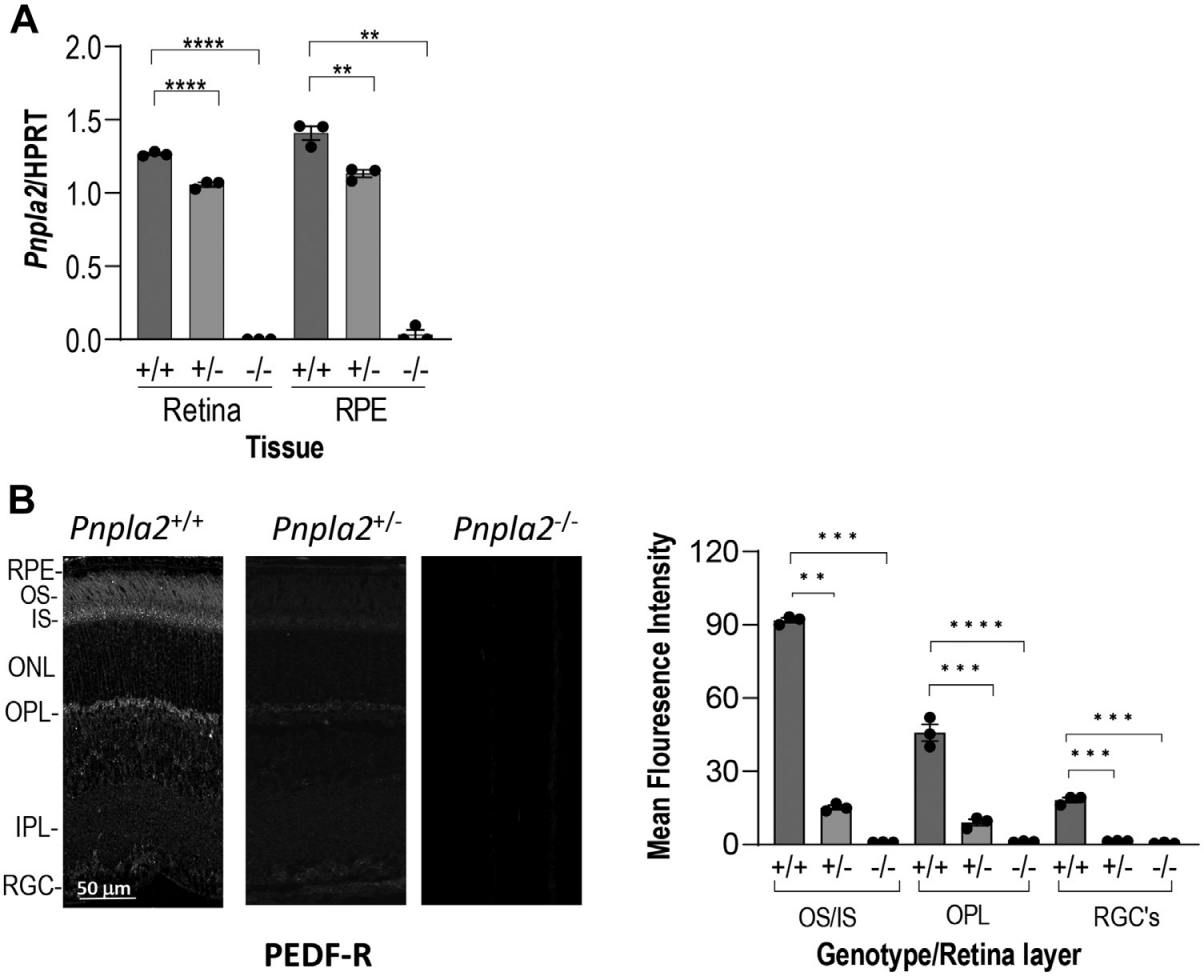
*Pnpla*2 mRNA, and PEDF-R distribution in the retina. A: *Pnpla2* expression (relative to *HPRT*) in RPE, and retina from 3 months old *Pnpla2*^*+/−*^ and *Pnpla2*^*−/−*^ and *Pnpla2*^*+/+*^ mice. Each data point corresponds to the average of three PCR reactions per tissue, indicated in the x-axis, *N* =3 per group. B: Representative fluorescent micrographs of PEDF-R (green) from retinas of *Pnpla2*^*+/+*^, *Pnpla2*^*+/−*^, and *Pnpla2*^*−/−*^ mice at 3 months of age. For all immunofluorescences shown three retinas per group and two sections per retina were evaluated using Dunette’s multiple comparisons, One-way ANOVA. ∗∗*P* < 0.001, ∗∗∗∗*P* < 0.00001.

We determined the effects of the CRISPR *Pnpla2* gene deletion on transcriptional repression of *Pnpla2* by performing RT-PCR on RNA isolated from the whole retina and isolated RPE/choroid tissues. As shown in Fig. 2A, the relative levels of *Pnpla2* mRNA decreased with gene deletion from the *Pnpla2*^+/+^ WT control (1.26 ± 0.01) to the heterozygous *Pnpla*2^+/−^ (1.05 ± 0.01), being undetectable in the homozygous *Pnpla*2^−/−^ (0.00 ± 0.00) (Ordinary one way-ANOVA *P*-value <0.0001). Similarly, the levels of *Pnpla2* transcripts decreased with gene deletion in the RPE (*Pnpla2*^*+/+*^, 1.40 ± 0.04; *Pnpla2*^*+/−*^, 1.13 ± 0.02; and *Pnpla2*^*−/−*^, 0.03 ± 0.03) and in other organs (see supplemental Fig. S1). Thus, a successful depletion of *Pnpla2* transcripts in the retina and other tissues was achieved upon CRISPR *Pnpla2* gene deletion.

**Fig. 4:**
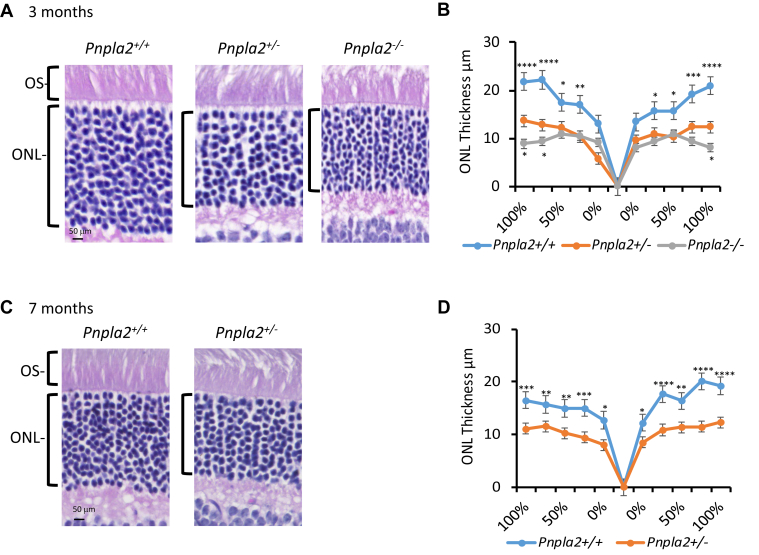
Histological evaluation of the retina of the *Pnpla2-KO* mouse line. A and C: Microphotographs of retina sections of 3- and 7-month-old *Pnpla2*^*+/−*^, *Pnpla2*^*−/−*^, and *Pnpla2*^*+/+*^ mice stained with hematoxylin and eosin. B and D: Spider plot analysis illustrating the thickness of the outer nuclear layer (ONL) of *Pnpla2*^*+/−*^, *Pnpla2*^*−/−*^, and *Pnpla2*^*+/+*^ mice. For all histology shown, three retinas per group and two sections per retina were evaluated and each data point corresponds to the average ± SEM per location relative to the optic nerve per genotype by either using Dunette’s multiple comparison, One-way ANOVA for 3 months group and unpaired *t* test for 7 months group. ∗*P* < 0.05, ∗*P* < 0.001, ∗∗∗*P* < 0.0001, ∗∗∗∗*P* < 0.00001.

Next, we examined the distribution of the PEDF-R protein in the mouse retinas and observed PEDF-R immunoreactivity in the photoreceptor outer segments, inner segments, and the outer plexiform layer layers in the *Pnpla2*^*+/+*^ (Fig. 2B, C). PEDF-R was also detected in the retinal pigment epithelium (RPE). We have reported previously that *Pnpla2* transcripts are expressed in all nuclear layers of mouse neural retinas and RPE (13), in agreement with the distribution of the PEDF-R protein in the mouse retina. The intensity of the immunolabelling was considerably decreased in the *Pnpla2*^*+/−*^ and undetectable in *Pnpla2*^*−/−*^ retinas (Fig. 2B, C). We also noticed a decrease in retinal outer nuclear layer (ONL) thickness in the retinas of PEDF-R-deficient mice compared to the littermate controls, suggesting that PEDF-R deficiency affected the morphology of the retina and in particular, the photoreceptors (see quantification of ONL height described below in Fig. 4). Nevertheless, these observations indicated that ablation of the *Pnpla2* gene resulted in the depletion of PEDF-R from the photoreceptors.

### *Pnpla2* knockdown altered the retinal morphology

**Fig. 3:**
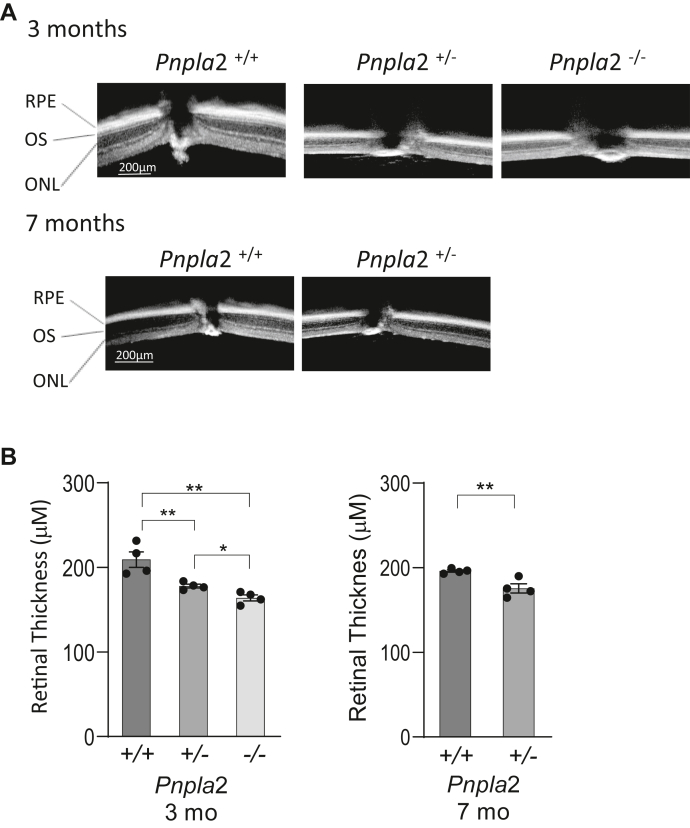
Retinal thickness determined by OCT. A: Representative images of whole retinas from the in vivo OCT scan of *Pnpla2*^*+/+*^, *Pnpla2*^*+/−*^, and *Pnpla2*^*−/−*^ mice at 3 months and *Pnpla2*^*+/+*^ and *Pnpla2*^*+/−*^ at 7 months of age are shown as indicated. B: Histograms showing the retinal thickness (*y-axis*) of *Pnpla2*^*+/+*^, *Pnpla2*^*+/−*^, and *Pnpla2*^*−/−*^ mice (*x-axis*). For all OCT shown, four retinas per group were evaluated and each data point corresponds to the thickness of one retina genotype by either using Dunette’s multiple comparisons, One-way ANOVA for 3 months group and unpaired *t* test for 7 months group. ∗*P* < 0.05, ∗∗*P* < 0.001.

Cross-sectional images of the mouse retinas were obtained by spectral domain SD-OCT, a non-invasive imaging method that assesses the morphology of the retina in vivo. Figure 3 shows representative tomographic retinal images of *Pnpla2*^*+/+*^, *Pnpla2*^*+/−*^*,* and *Pnpla2*^*−/−*^ mice. We measured the total retinal thickness between the inner edge of the retina and the RPE per quadrant, and the measurement of retinal thickness was reiterated in each quadrant. We observed similar retinal thickness among all quadrants of the same mouse, indicating that the measurement was homogeneous throughout each retina and that retinal thickness changes did not preferentially affect a specific quadrant but the whole retina. Comparing the retinal thickness among different mice, we found that the retinal thickness of 3-month-old mice with *Pnpla2* gene deletions was significantly smaller than those of *Pnpla2*^*+/+*^ control mice (*Pnpla*2^−/−^ 163.9 ± 3.52, *Pnpla2*^+/−^ 177.9 ± 2.156, and *Pnpla2*^*+/+*^ 209.2 ± 9.12, with one-way ANOVA ∗∗*P* < 0.0075 for *Pnpla2*^*+/−*^ and ∗∗∗*P* <0.0007 for *Pnpla*2^−/−^). At 7 months of age, the heterozygous mice also had smaller retinal thickness than those of control mice (*Pnpla2*^*+/−*^ 175.5 ± 5.31, and *Pnpla2*^*+/+*^ 203.7 ± 4.60, unpaired *t* test ∗∗*P* < 0.007). We concluded that the *Pnpla2* deletions negatively affected the retinal thickness (Fig. 3), which is consistent with the differences in the retinal thickness observed by immunofluorescence for *Pnpla*2^−/−^ and *Pnpla*2^+/+^ retinas in Fig. 2B (above).

Histological examination of cross-sections of retinas revealed thinner ONL in *Pnpla2*^*−/−*^ and in *Pnpla2*^*+/−*^ mice than the ONL of their littermate controls at both 3 and 7 months of age (Fig. 4A, C). We noted that all the layers of the retina were present in all genotypes. The ONL thickness from all groups was measured at five definite points on both the superior and the inferior sides of the optic nerve head to obtain the spider plotting graphs shown in Figure 4B, D. The spider plots showed that *Pnpla*2^+/−^ and *Pnpla*2^−/−^ mice possess thinner ONL on both sides of the retina when compared to *Pnpla*2^+/+^ littermates at 3 and 7 months of age (one-way ANOVA with Bonferroni posttests compared KO mice with *Pnpla*2^+/+^ in all segments, ∗∗∗*P*< 0.0001, ∗∗*P*< 0.001, ∗*P*< 0.01).

**Fig. 5:**
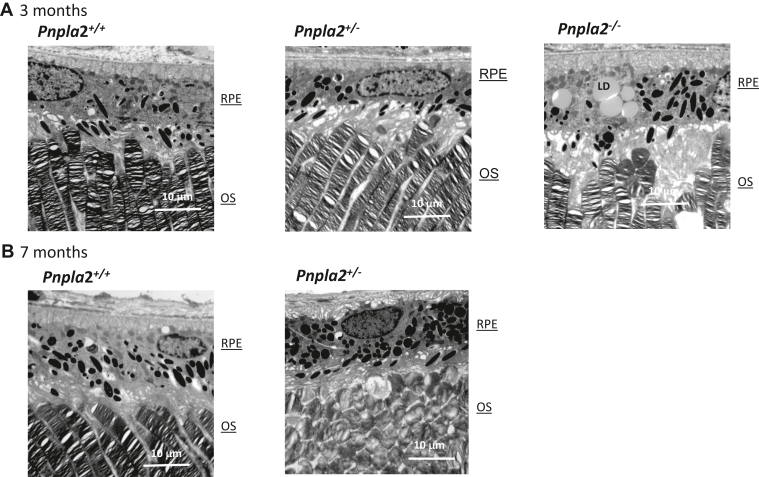
Transmission electron microscopy of retinas of the *Pnpla2-KO* mouse line. A and B: Representative transmission electron micrographs of *Pnpla2*^*+/+*^*, Pnpla2*^*+/−*^*,* and *Pnpla2*^*−/−*^ mice at 3 months of age and *Pnpla2*^*+/+*^*, Pnpla2*^*+/−*^ at 7 months of age. Scale bar: 10 μm. The representative images were selected after examinations of micrographs from four eyes (n*|*N = 4) of each genotype, *Pnpla2*^*−/−*^, *Pnpla2*^*+/+*^*,* and *Pnpla2*^*+/−*^ at 3 and 7 months of age.

The retinas of the three cohorts of mice, *Pnpla*2^+/+^, *Pnpla*2^+/−^, and *Pnpla*2^−/−^, all at 3 months of age and a group of the first two at 7 months old were compared at the ultrastructural level using a transmission electron microscope. At 3 months old, the photoreceptor OS of the *Pnpla*2^+/+^ and *Pnpla*2^+/−^ mice had a normal appearance, having membrane disks mostly regularly stacked, while the ones of the *Pnpla2*^*−/−*^ mice were unevenly arranged and their membrane disks were slightly irregularly stacked (Fig. 5A). Similarly, at 7 months, the OS of *Pnpla*2^+/+^ mice had a normal appearance with membrane disks mostly regularly stacked; however, those of *Pnpla*2^−/−^ mice were irregular in shape and showed signs of degeneration (Fig. 5B). The heterozygous and homozygous *Pnpla*2 KO mice also showed enlargement of the apical processes’ region of the RPE (Fig. 5A, B) (quantification of apical processes *Pnpla2*^*+/+*^, 2.5–7.5 μm, *Pnpla2*^*+/−*^; 5–12.5 μm; and *Pnpla2*^*−/−*^, 10–25 μm); furthermore, there were aberrations in the basal infoldings of the RPE (images not shown). Only the homozygous *Pnpla*2^−/−^ KO mice showed an accumulation of lipid deposits in the RPE cytoplasm, and some were in large clusters (Fig. 5A, right image).

### *Pnpla2* knockdown decreased PLA_2_ activity in the retina

**Fig. 6:**
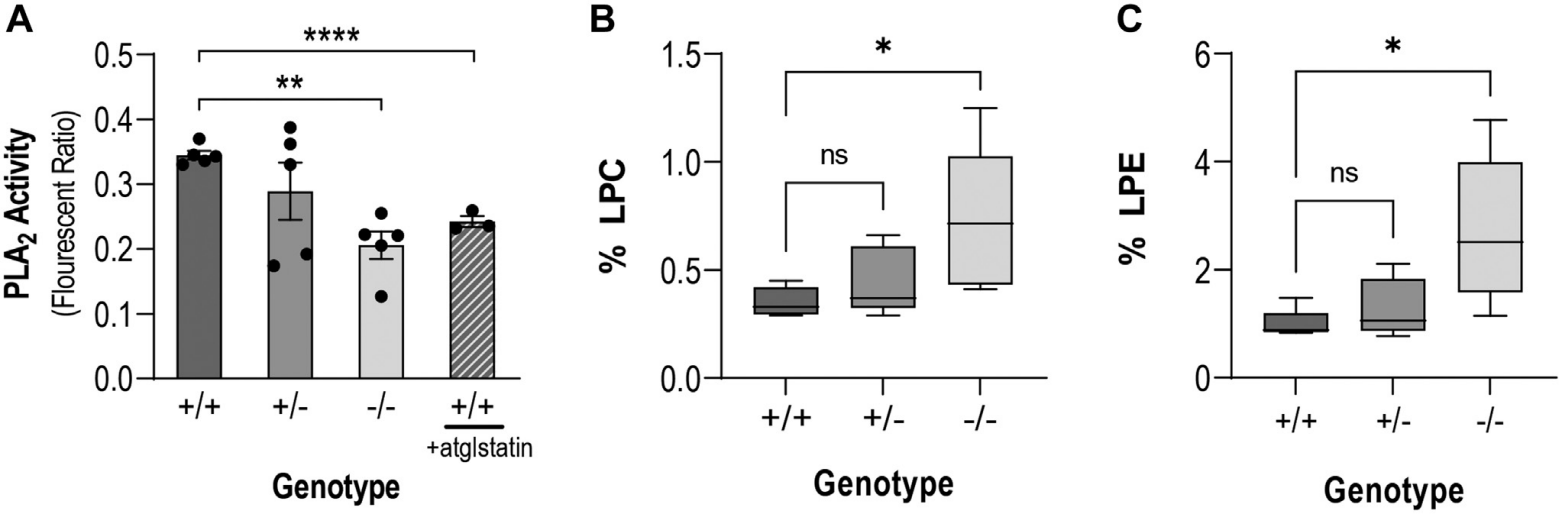
Phospholipase A2 and phospholipids in the retina of the Pnpla2- KO mouse. A: Retinal phospholipase A2 activity was measured in retinal extracts from *Pnpla2*^*+/+*^, *Pnpla2*^*+/−*^, and *Pnpla2*^*−/−*^ mice at 3 months of age (solid color bars) and in retinal extracts of *Pnpla2*^*+/+*^ mice at 3 months of age supplemented with 3.5 μM of atglistatin (hatched bar). For the experiments, three to five retinas were used per group, and each data point corresponds to one retina. *N* = 3–5. B and C: Phospholipids composition analyzed by mass spectrometry in whole retinas of *Pnpla2*^*+/+*^*, Pnpla2*^*+/−*^, *and Pnpla2*^*−/−*^ mice showing accumulation of LPC-DHA (B) and LPE -DHA (C) in percentage to other phospholipids. *n* = 3 retinas per genotype by either using Dunette’s multiple comparison, One-way ANOVA (analysis of variance) for 3 months group and unpaired *t* test for 7 months group. ∗*P* < 0.05, ∗∗*P* < 0.001, *∗∗∗∗P*< 0.00001, ns = not significant.

We assessed the PLA_2_ activity in the retinas of mice of the three genotypes. Suspension of *Pnpla2*^*+/+*^ retinal extracts exhibited measurable PLA_2_ activity (0.35 ± 0.01, which decreased in *Pnpla*2^*−/−*^ retinas (0.21 ± 0.02) (Fig. 6A). Given that there was residual activity in the retina of PEDF-R-deficient mice, we assumed that it was due to PLA_2_ activities unrelated to PEDF-R. Therefore, we assayed the *Pnpla2*^*+/+*^ retinas in the presence of a specific inhibitor of PEDF-R, atglistatin, to inhibit the PEDF-R enzyme activity exclusively. We found that atglistatin lessened the PLA_2_ activity of *Pnpla2*^*+/+*^ matching the activity of *Pnpla2*^*−/−*^ retinas. These observations imply that the residual PLA_2_ enzymatic activity in retinal extracts from mice lacking PEDF-R and retinal extracts from WT mice supplemented with the specific PEDF-R inhibitor was due to PLA_2_ activities unrelated to that of PEDF-R in the retina. Thus, PEDF-R loss caused a specific decrease in PLA_2_ activity in the *Pnpla*2^*−/−*^ mouse retina.

### Phospholipids in the retina

We hypothesized that loss of PEDF-R phospholipase function in the retina affects the retinal glycerophospholipid composition. We, therefore, determined the phospholipid composition of retinas of the three genotypes with a particular focus on the main retinal phospholipids PC, PE, and PS using LC/MS/MS. Compared to *Pnpla*2^+/+^ and *Pnpla*2^+/−^ mice, we observed increased levels of lysophosphatidyl choline-DHA and lysophosphatidyl ethanolamine-DHA in the retina of *Pnpla*2^−/−^ mice at 3 months of age (Fig. 6B, C). However, we did not detect the presence of phosphatidylserine (PS)-containing DHA in the retina of these mice at this age, although other PS lipids were present. In addition, the total fatty acid analyses did not show significant changes in the fatty acid profile of the retina of the *Pnpla2* KO mice compared to WT controls. These findings suggest that remodeling of phospholipid composition occurred in the PEDF-R-deficient retina occurred, favoring the presence of fatty acid 22:06-containing species and that DHA esterified at the *sn-2* position in the retinal PC and PE are likely physiological ester-bond targets for release of DHA by PEDF-R in photoreceptors.

### *Pnpla2* deletion decreases photoreceptor visual pigment markers

**Fig. 7:**
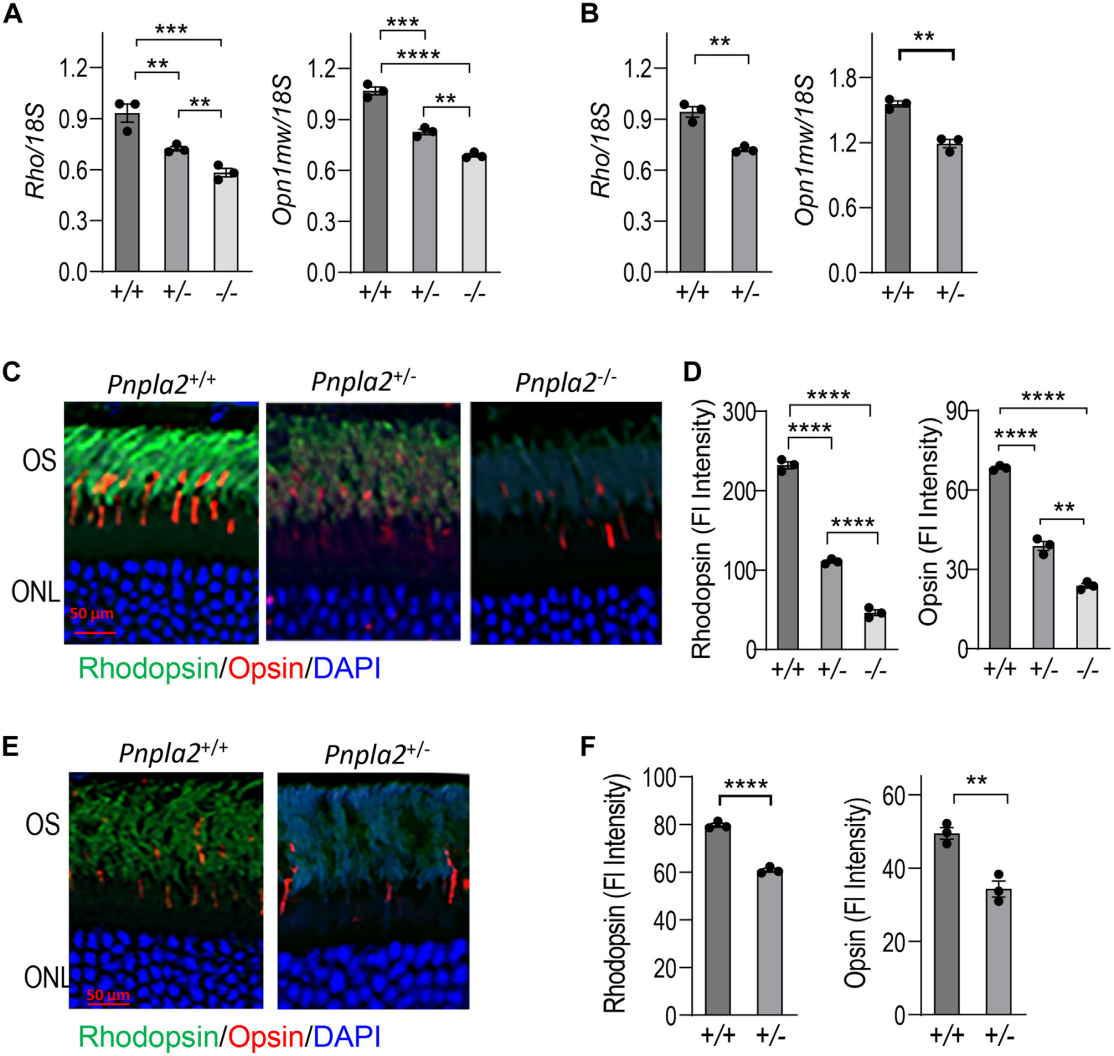
Effects of *Pnpla2* deletion on photoreceptor markers, rhodopsin, and opsin. A and B: Levels of rhodopsin and opsin gene expression in the retinas of *Pnpla2* animals was determined by RT-PCR using 18S as a housekeeping gene at 3 months (A) and 7 months (B) of age. For all RT-PCR shown, n = 3 retinas per group. C and E: Representative fluorescent micrographs of retinas stained with antibodies to rhodopsin (green) and opsin (red) from *Pnpla2*^*+/+*^*, Pnpla2*^*+/−*^ and *Pnpla2*^*−/−*^ mice at 3 months (C) and 7 months (E) of age. D and F: Quantification of immunofluorescence of rhodopsin and opsin markers was performed using ImageJ. For all immunofluorescence analyses shown 3 retinas were used per group and two sections per retina, and each data point corresponds to the average of one retina, by either using Dunette’s multiple comparison, One-way ANOVA for 3 months group and unpaired *t* test for 7 months group. ∗∗*P* < 0.001, ∗∗∗*P* < 0.0001, ∗∗∗∗*P* < 0.00001. Scale bar indicates 50 μm.

The rhodopsin and opsin proteins are embedded in membranes surrounded by phospholipids in the outer segments. A change in phospholipid composition may also affect the pigment composition in the photoreceptors. The mRNA levels of rhodopsin (*Rho*) and opsin (*Opn1mw*) genes in the retina decreased with the *Pnpla2* gene deletion in *Pnpla*2^−/−^ and in *Pnpla*2^+/−^ retinas. The distinct distribution of the *Rho/18S* ratios (0.6 ± 0.02), and the *Opn1mw/18S* ratios (0.69 ± 0.01) of the homozygously deleted retinas were lower than the ones for the heterozygous retinas (*Rho/18S*, 0.72 ± 0.01 and *Opn1mw/18S*, 0.83 ± 0.01) and *WT* control retinas (*Rho/18S*, 0.93 ± 0.05and *Opn1mw/18S*, 1.1 ± 0.02) at 3 months of age (Fig. 7A). Similarly, decreases were noticed at 7 months of age for heterozygous *Pnpla*2^+/−^ retinas (*Rho/18S* 0.72 ± 0.01 and *Opn1mw/18S* 1.2 ± 0.03) when compared to *Pnpla2*^+/+^ control retinas (*Rho/18S* 0.94± 0.01 and *Opn1mw*/18S 1.6 ± 0.02) (Fig. 7B).

To confirm that the changes in mRNA expression levels in the three genotypes correlated with changes in protein expression, we assessed the distribution of the rhodopsin and opsin proteins in photoreceptors of retinal sections of the *Pnpla2* knockout mice and controls. Immunofluorescence intensities decreased for both pigments in the homozygous mice at 3 months of age and in the heterozygous mice at 3 and 7 months of age when compared to WT control littermates (Fig. 7C, E). Quantification of the fluorescence indicates that the intensities of rhodopsin and opsin of heterozygous mice at 3 months old were about half of the WT control littermates (rhodopsin 0.47-fold and opsin 0.57-fold). Rhodopsin and opsin in the retinas of the homozygously deleted mice were even lower, with about one-fourth of the controls (rhodopsin 0.2-fold and opsin 0.35-fold). The retinal rhodopsin and opsin levels of controls at 7-month-old were lower than at 3-month-old (compare Fig. 7D, F), which declined in the heterozygous at 7 months of age (rhodopsin 0.76-fold, opsin. 0.68 of the controls) (Fig. 7D).

**Fig. 8:**
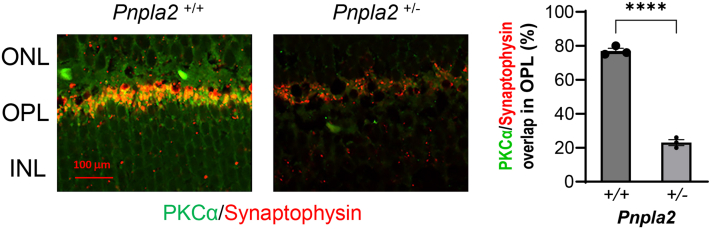
Effects of *Pnpla2* deletion on PKC-α and synaptophysin. Representative fluorescent micrographs of overlapping of anti-PKC-α (green) and anti-synaptophysin anti-PKC-α (red) in retinas of *Pnpla2*^*+/+*^*, Pnpla2*^*+/−*^, and *Pnpla2*^*+/−*^ mice (left side). Quantification of overlapping of anti-PKC-α and anti-synaptophysin markers using ImageJ (right side). For all immunofluorescence analyses shown 3 retinas were used per group and 2 sections per retina and each data point corresponds to the average of one retina by doing an unpaired *t* test. *∗∗∗∗P* < 0.00001. Scale bar indicates 100 μm.

Given that bipolar cells receive inputs from photoreceptor cells making direct synaptic contacts for the visual process, we performed immunohistochemistry to examine bipolar cells in heterozygous mice relative to controls at 7 months of age. We co-labeled retinal cross-sections with anti-PKCα, a marker for rod bipolar cells, and anti-synaptophysin, a marker for the photoreceptor ribbon synapse. We found that PKCα and synaptophysin staining in WT mice overlapped, showing that rod bipolar cells extended dendrites to the photoreceptor terminals (Fig. 8). In contrast, in heterozygous retinas, there was no overlap of the two markers, indicating that the rod bipolar cell dendrites were retracted toward their cell bodies (Fig. 8). To determine changes in dendritic overlap with the photoreceptor synaptic terminals, we quantified the extent of double labeling of the two antibodies. We found a significant decrease in the overlap of fluorescence intensity between the two antibodies with an overlap mean for *Pnpla*2^+/−^ of 23 ± 1.8% and for *Pnpla2*^+/+^ of 77± 1.5%. These findings implied that loss of PEDF-R impacted negatively on the visual process.

### PEDF-R deficiency causes photoreceptor cell death

**Fig. 9:**
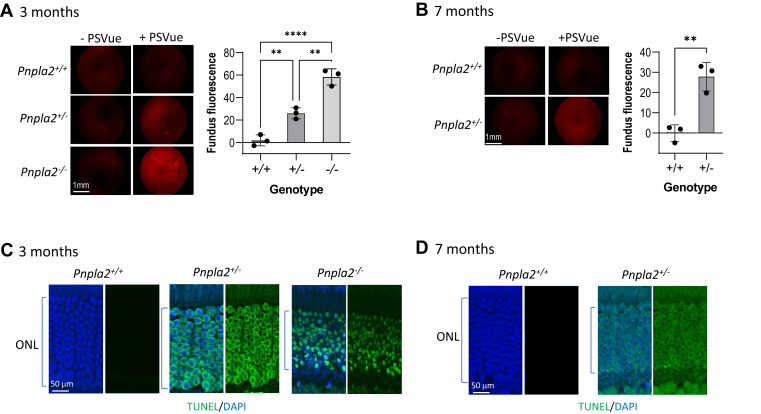
PEDF-R deficiency causes photoreceptor cell death. A and B: Fluorescence angiography micrographs of retinas at 3 and 7 months old of *Pnpla2*^*+/−*^ and *Pnpla2*^*−/−*^ and *Pnpla2*^*+/+*^ mice with or without PSVue® as indicated (left side). Scale bar indicates 1 mm of the mouse eye. Plots of the quantification of fluorescence intensity using ImageJ are shown to the right of the micrographs (right side). For all fluorescence intensity images shown 3 retinas per group and 3 images per retina were used and each data point corresponds to the average of one retina, by either using Dunette’s multiple comparison, One-way ANOVA for 3 months group and unpaired *t* test for 7 months group. ∗∗*P* < 0.001, *∗∗∗∗P* < 0.00001. C and D: Representative fluorescent micrographs of staining with TUNEL (green) and DAPI (blue) of retinas of *Pnpla2*^*+/+*^*, Pnpla2*^*+/−*^and *Pnpla2*^*−/−*^ mice at 3 and 7 months of age are shown, as indicated. For all assays shown, three retinas per group and two sections per retina were evaluated. Scale bar indicates 50 μm.

The effects of the ablation of PEDF-R on photoreceptor survival were examined. First, we assessed cell death using PSVue-550, a small molecule probe that binds PS residues exposed on the surface of cells undergoing cell death by apoptosis in the retina (26). Eyedrops of the fluorescent probe were administered to the eyes of our *Pnpla2* KO mice, and detection of the fluorescence of the surface exposed PS was imaged in the fundi 24 h post in vivo administration. Figure 9A and B show greater fluorescence intensities in the fundi of the heterozygous and even more in homozygously deleted eyes that received the probe than their contralateral eyes treated with eyedrops of only vehicle, HBSS, the dilutant of the PSVue-550 probe. WT control mice did not have detectable fluorescence in their fundi. Quantification confirmed higher fluorescence intensity in heterozygous and homozygously deleted mice relative to control animals at 3 months of age (Ordinary one-way ANOVA *P*-value <0.0001 between *Pnpla2*^*−/−*^ and *Pnpla2*^*+/*+^ and *Pnpla2*^*+/−*^ *P*-value <0.001 when compared to *Pnpla2*^*+/+*^) (Fig. 9A). Similarly, fluorescence intensity was higher for heterozygous than control littermates at 7 months of age (unpaired *t* test *P*-value < 0.0001 between *Pnpla*2^+/−^ and *Pnpla2*^+/+^) (Fig. 9B). These observations demonstrated that the mice deficient in PEDF-R had PS surface exposed in retinal cells. Then, we performed TUNEL assays on retinal sections of all genotypes. While TUNEL-positive cells were mainly absent in the WT control sections, they were detectable in the ONL of the heterozygous and homozygous mice at 3 months of age (Fig. 9C) and in heterozygous mice at 7 months of age (Fig. 9D). We noticed that in *Pnpla*2^−/−^ the nuclei in the ONL appeared pyknotic. TUNEL-positive cells were also observed in the inner nuclear layer, retinal ganglion cell layer, and most importantly in the ONL (not shown). Altogether, the findings imply that the removal of PEDF-R triggered a cell death program in photoreceptors.

### PEDF-R loss causes alterations in retinal function

**Fig. 10:**
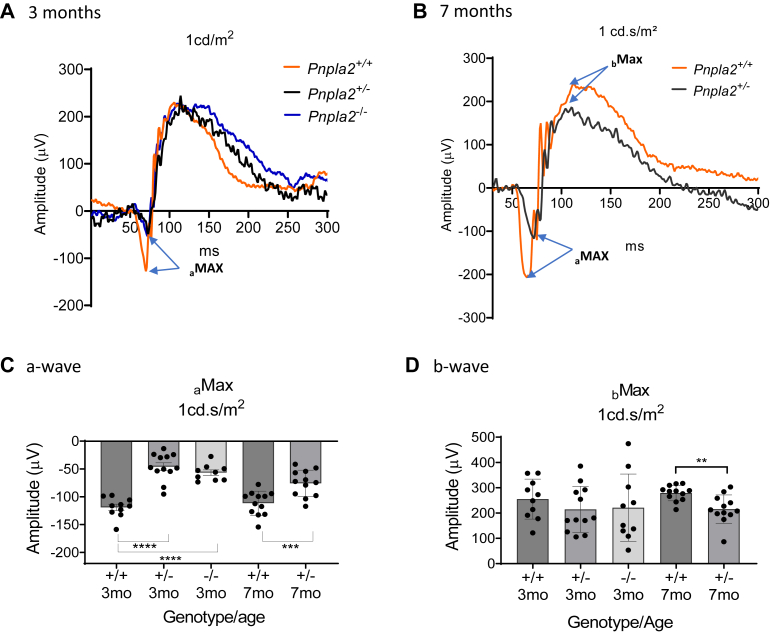
PEDF-R deficiency causes alterations of retinal function. A and B: Representative ERG waveforms for 3-month-old and 7-month-old mice, respectively, show amplitude (*y*-axis) as function of time in milliseconds (ms, *x*-axis). C and D: Graphs showing photoreceptor a-wave amplitudes and b-wave amplitudes, respectively, at 1 cd.s/m^2^ of each genotype and age of mice. For ERG data shown, the number of mice evaluated were n = 10 for *Pnpal2*^*+/+*^, n = 12 for *Pnpal2*^*+/−*^ and n = 10 for *Pnpal2*^*−/−*^mice at 3 months of age, by using Dunette’s multiple comparison, One-way ANOVA; and n = 12 for *Pnpal2*^*+/+*^ and n = 12 for *Pnpal2*^*+/−*^ mice at 7 months of age, by doing an unpaired *t* test. ∗∗*P* < 0.001, ∗∗∗*P* < 0.0001 *∗∗∗∗P*< 0.00001.

The observed dysmorphology of the *Pnpla2*^*−/−*^ photoreceptors led us to also assess retinal function by electroretinography. Figure 10A and B show representative electroretinograms (ERGs) for the *Pnpla*2^−/−^, *Pnpla2*^*+/−*^, and *Pnpla2*^*+/+*^ mice at 3 months and 7 months of age, respectively. ERGs of more than 9 animals showed that the mean amplitude of the a-wave (a_max_) of *Pnpla2*^*−/−*^ (54.94 ± 4.67 V) and *Pnpla2*^*+/−*^ (45.98 ± 7.05 μV) were lower than *Pnpla2*^*+/+*^ controls (119.1 ± 5.72 μV) at 3 months of age, and the differences were statistically significant (Ordinary one way-ANOVA *P*-value <0.001 between *Pnpla2*^*−/−*^, *Pnpla2*^*+/−*^ when compared to *Pnpla2*^*+/+*^) (Fig. 10C). Similarly, *Pnpla2*^*+/−*^ mice at 7 months of age had lower a_max_ amplitude (75.80 ± 6.82 μV) when compared to *Pnpla2*^*+/+*^ (111.6 ± 6.24 μV) and the difference was statistically significant (unpaired *t* test *P*-value 0.0008 between *Pnpla2*^*+/−*^ and *Pnpla2*^*+/+*^) (Fig. 10C). Thus, a reduced a_max_ amplitude for the *Pnpla2* knockdown retinas was indicative of the requirement of *Pnpla2* in photoreceptor function.

There were no significant differences in the b-wave amplitude (b_max_) among *Pnpla*2^−/−^, *Pnpla*2^+/−^, and *Pnpla*2^+/+^ mice at 3 months of age, as shown in Fig. 10D. However, when the b-wave (b_max_) was measured at 7 months of age, we noticed a statistical difference in amplitude between the heterozygous *Pnpla*2^+/−^ (215.5 ± 16.5 μV) and control *Pnpla*2^+/+^ (279.1 ± 8.91 μV) mice (unpaired *t* test *P*-value 0.0023 between *Pnpla*2^+/−^ and *Pnpla2*^+/+^). The ERG b-wave changes agreed with the lack of visual function based on decreases in bipolar/photoreceptor synapse ribbon markers, shown above (see Fig. 8), implying that that the photoreceptor-mediated inner retinal responses manifest with age after 3 months of age, such as at 7 months after birth.

However, there were no statistical differences in photopic, light-adapted ERG a-wave and b-wave among *Pnpla*2^−/−^, *Pnpla*2^+/−^, and *Pnpla*2^+/+^ mice at 3 months and 7 months of age (supplemental Fig. S3). Altogether, the electrophysiological results imply that the removal of PEDF-R negatively affected rod-driven responses rather than cone-driven responses as well as those for the RPE.

## Discussion

Our study has revealed that loss of global PEDF-R function disrupts lipid homeostasis and causes the degeneration of mouse photoreceptor neurons. Deletion of only one allele of the PEDF-R encoding gene *Pnpla2* reduces the ONL thickness, which is accompanied by alterations in phospholipid composition, deterioration in photoreceptor morphology and synaptic contacts at the OPL, and reduction of visual pigments rhodopsin and opsin, along with light-independent photoreceptor apoptosis in mice. Moreover, the degeneration of photoreceptors is more pronounced when both alleles are deleted in the homozygous *Pnpla2 null* mice, resulting in significant functional and ultrastructural changes in the retina, for example, reduced retinal function likely due to loss of photoreceptor cells in mice. It is clear that there are other phospholipases in the retina other than PEDF-R (as shown by differential pharmacological sensitivity), but apparently, they cannot replace the proposed essential function provided by PEDF-R. The conclusions agree with studies demonstrating that pharmacological inhibition of PEDF-R phospholipase activity attenuates corneal nerve regeneration (16). Additionally, the results on the increase in the levels of retinal lysophospholipids PC22:6 and PE22:6 are in line with previous studies showing that dysregulation of phospholipid metabolism leads to photoreceptor cell death and retinal degeneration in the *rd11* retinal degeneration model (30, 31). These findings identify PEDF-R as an important component for photoreceptor structure and visual activity, highlighting its role in phospholipid metabolism for retinal survival and function.

Most studies on the *PNPLA2* gene have focused on the triglyceride lipase activity of PEDF-R protein, also called ATGL (adipose triglyceride lipase) or desnutrin, using extraocular biological systems that are rich in triglycerides contained in lipid droplets. As far as we know, a constitutively *Atgl*-KO mouse and mice with ablated desnutrin/*Pnpla2* targeted to brown adipose tissue and pancreatic β cells have been generated (29, 32, 33). However, the presence of frequent mutations in the *rd8* gene that causes retinal degenerations in vendor mice and embryonic stem cells (28) precluded the use of these existing mice in an experimental design for studies of the retina. Recently, we generated a cKO mouse with a targeted deletion of the *Pnpla2* gene in the RPE, which is in a retinal degeneration *rd8*-free background (20). The deletion causes the formation of large lipid deposits with a delay in the digestion of the phospholipid-enriched photoreceptor OS during phagocytosis by the RPE cells but without obvious photoreceptor or retinal degeneration phenotypes (20). Our efforts to generate a conditional cKO mouse for photoreceptors were not successful due to the lack of a specific photoreceptor promoter that is not leaky. Nevertheless, like the *Atgl-*KO mice (29), the constitutively *Pnpla2*-KO mice generated here have a decrease of plasma-free fatty acids, triglycerides, and β-hydroxybutyrate, no significant changes in cholesterol levels in serum (see supplemental Fig. S1), and die at 3–4 months of age likely due to increase lipid accumulation in the heart. Thus, our PEDF-R-deficient mice are in a retinal degeneration-free background and prove useful for studies of the retina.

The neural retina offers an exceptional system for the investigation of the phospholipase action of PEDF-R/PNPLA2/ATGL because phospholipids constitute a major fraction of the total retinal lipid content, and the source of triglycerides is minimum in photoreceptors given that the photoreceptor cell layer is free of vessels (3, 34). Phospholipids play a vital role in the cellular structure and physiology of the photoreceptors by forming the lipid bilayers that maintain cell boundaries as well as serving as an energy reservoir and precursors for downstream signaling molecules (35). The photoreceptors contain the highest amounts of DHA-rich phospholipids in the human body (36). It is known that the activation of DHA-mediated downstream survival signaling in cultured photoreceptor cells requires the release of DHA from phospholipids of cellular membranes (35), implying that the PEDF-R phospholipase is likely required for a sensing task. PEDF-R acts to catalyze phospholipids from cellular membranes into bioactive fatty acids, such as DHA with neurotrophic and cell survival properties (12, 16, 37, 38, 39). PEDF-R may also serve to modify the fatty acid composition of phospholipids along with lysophospholipid acyltransferase families of enzymes. The data suggest that while the PEDF-R catalyzes phospholipid substrates in the WT retinas, they accumulate in the ONL of the *Pnpla2*-KO mice without PEDF-R phospholipase. Consistent with the lipid accumulation phenotypes, phospholipid analyses reveal retinal buildup of LPCs lysophospholipid PC22:6 and PE22:6 when PEDF-R declines and implies that these species are likely physiological phospholipid substrates for PEDF-R in the photoreceptors. These observations suggest that in a probable mechanism to activate the DHA-mediated downstream survival signaling in photoreceptors, PEDF-R acts to release 22:6 from these phospholipids, which its agonist, PEDF, can activate. In this way, the PEDF-R/PEDF axis modulates the retinal lipidome and in turn, supports survival and visual function.

Our conclusions regarding a relationship between lipid metabolism and neuronal functions agree with previous studies showing lipid metabolism dysregulation and photoreceptor degeneration in the *Drosophila* retina caused by genetic deletion of the photoreceptor-specific miR-210 and by mutations of *Pnpla6*—linked to childhood blindness—in which photoreceptor cell death associates with the buildup of lysophosphatidylcholine and lysophosphatidic acid (40, 41). Similarly, they agree with many reports demonstrating that lipid homeostasis is necessary for maintaining neuronal function and synaptic plasticity in the central nervous system (42, 43). Our findings support the idea that PEDF-R is a link between phospholipids and photoreceptor survival and function.

Although the PEDF-R-deficient photoreceptors exhibit signals known as hallmarks of apoptosis (*eg*, cell surface PS exposure, DNA fragmentation, cell shrinking, and nuclear condensation), loss of photoreceptor cells is not evident. A possible explanation for these observations is that the *Pnpla2*^*−/−*^ photoreceptor cells might not have activated still unknown required molecular components to proceed with the death program at 3–7 months of age. The delay in digesting photoreceptor OS by the PEDF-R-depleted RPE (20) could trigger the initial steps of a cell death program in photoreceptors without proceeding to the final steps for their complete demise. The observations raise interesting questions regarding the association between cell death biomarkers and cell loss to be addressed in the future. Our findings show a slow photoreceptor degeneration associated with PEDF-R-deficiency and are in line with a mouse model with mutations in the gene for progressive rod-cone degeneration that causes dysmorphologies of photoreceptors with slow reduction of ONL height in mice between 3 weeks and 17 months of age (44). Interestingly, while the homozygous *Pnpla2*^*−/−*^ and heterozygous *Pnpla2*^*+/−*^ mice have visual functional defects, only the homozygous has noticeable lipid deposits in the RPE. The results imply that the lipid accumulation phenotype in the RPE is unrelated to the photoreceptor malfunctioning, deformities, and death seen in our *Pnpla2* KO mice and suggest that *Pnpla2* deletion in the photoreceptors may suffice for the photoreceptor degenerative phenotype defect. In this regard, we reported previously that deletion of *Pnpla2* targeted only in the RPE of mice resulted in lipid deposits in the RPE cells without an apparent photoreceptor degeneration (20). It also argues that the lysophospholipids or the liberated fatty acids generated by the PEDF-R/PNPLA2 activity in the RPE cell have minimal, if any, importance to the neighboring, underlying photoreceptor cells. It is worth highlighting that, regardless of the fact that PEDF-R was globally ablated, the pathological consequence observed in the mouse model we generated for the present study was largely due to changes in the photoreceptor cells per se.

Moreover, mutations of the *PNPLA*2 gene in humans cause neutral lipid storage disease with myopathy (NLSD-M), a very rare condition characterized by the accumulation of cytoplasmic triglyceride droplets in various tissues and mainly associated with skeletal and cardiac muscle disease (45, 46). Involvement of ocular, ophthalmological, or visual defects in NLSD-M are not reported to date, suggesting some differences between human and mice lacking PEDF-R and/or, considering our findings in mouse retinal physiology, that a decrease in PEDF-R levels may pose a risk leading to slow progression of retinal degeneration.

In summary, the findings demonstrate a role for *PNPLA2* in photoreceptor survival and function and underscore phospholipid metabolism as a potential therapeutic target for some forms of blindness.

## Data availability

All data are contained within this manuscript.

## Supplemental data

This article contains supplemental data (47).

## Conflict of interest

none.
