# Supplementary material for: Ablation of pigment epithelium-derived factor receptor (PEDF-R/Pnpla2) causes photoreceptor degeneration
Source: J Lipid Res. 2023 Mar 17;64(5):100358. doi: 10.1016/j.jlr.2023.100358 (PMC10233210; doi:10.1016/j.jlr.2023.100358)
Supplement: Supplemental Figures [file mmc1.pdf]

SUPPLEMENTAL DATA

Results

Extraocular evaluation of the *Pnpla2*$^{-/-}$ mouse

Anatomical evaluation of several organs showed that the *Pnpla2*$^{-/-}$ mice had hearts that were enlarged and whiter in color when compared to *Pnpla*2$^{+/-}$ and *Pnpla2*$^{+/+}$ mice at 3 months of age (**Fig. S1B**). The liver, kidneys, and lungs were not drastically different among genotypes. The plasma free fatty acids (FAA) levels of 3-month old mice decreased with gene deletion from *Pnpla2*$^{+/+}$ (1.0 ± 0.03 nmol/µl) to *Pnpla*2$^{+/-}$ (0.81± 0.03 nmol/µl) and even lower for *Pnpla2*$^{-/-}$ (0.53 ± 0.06 nmol/µl) with a statistically significant difference (Ordinary one way-ANOVA *p*-value <0.02 between *Pnpla2*$^{+/+}$ and *Pnpla*2$^{+/-}$ and *p*-value <0.003 between *Pnpla2*$^{+/+}$ and *Pnpla2*$^{-/-}$) (**Fig. S1C)**. Similarly, plasma triglycerides (TG) levels of 3-month-old mice decreased with gene deletion from *Pnpla2*$^{+/+}$ (4.091 ± 0.28 mM), to *Pnpla2*$^{+/-}$ (2.05± 0.48 mM) and *Pnpla2*$^{-/-}$ (2.06 ± 0.23 mM) mice (Ordinary one way-ANOVA *p*-value <0.005 between *Pnpla2*$^{-/-}$, *Pnpla2*$^{+/-}$ when compared to *Pnpla2*$^{+/+}$). Plasma cholesterol levels among 6 mice of each genotype were not affected (*Pnpla2*$^{+/+}$, 94.72 ± 9.13 mg/dL), to *Pnpla2*$^{+/-}$ (104.8 ± 9.29 mg/dL) and *Pnpla2*$^{-/-}$ (109.01 ± 4.50 mg/dL). Lastly, plasma β-hydroxybutyrate levels of 3-month-old mice decreased with gene deletion from *Pnpla2*$^{+/+}$ (2.01 ± 0.15 mmol/L) to *Pnpla2*$^{+/-}$ (1.80 ± 0.12 mM) and *Pnpla2*$^{-/-}$ (0.51 ± 0.10 mM) mice (Ordinary one way-ANOVA *p*-value <0.005 between *Pnpla2*$^{-/-}$, *Pnpla2*$^{+/-}$ when compared to *Pnpla2*$^{+/+}$) (**Fig. S1C)**. These plasma parameters and anatomical observation in the heart matched those reported for the *Atgl*$^{-/-}$ mice (30).

Ocular evaluation of the *Pnpla2*$^{-/-}$

Optical coherence tomography angiography (OCTA) showed that the retinas of 3 months old *Pnpla2*$^{-/-}$ and *Pnpla2*$^{-+/-}$ mice at 3 and 7 months of age had white lesions located at the deeper levels of the retina and choroid**.** Examination of the ocular fundi showed the presence of an optic nerve disc pit, in

which a very small amount of retina tissue protrudes backwards through the defect in *Pnpla2*$^{-/-}$ at 3 months of age and *Pnpla2*$^{+/-}$ 3 and 7 months of age **(Fig. S2)**.

PEDF-R deficiency did not alter photopic retinal function. Graphs showing photoreceptor a-wave amplitudes and b-wave amplitudes, respectively, at 10cd.s/m^2 of each genotype and age of mice (**Fig. S3**). Photopic, light-adapted ERGs showed no apparent differences among genotypes at 3 and 7 months of age, implying that cone-responses were not affected by removal of PEDF-R. It suggests that removal of PEDF-R affects more negatively and selectively the rod photoreceptors under the conditions tested.

Materials and Methods

Free fatty acids, Cholesterol, Triglyceride and β-hydroxyburate quantification assay

After fasting the animals, a total of 50 µl of conditioned plasma samples were collected and used to quantify free fatty acids and triglycerides using the Free Fatty Acid Quantification Assay Kit (Colorimetric) (Abcam, catalog number ab65341), Triglyceride Assay Kit (Colorimetric) (Abcam, catalog number ab65336), Cholesterol Assay kit (Colorimetric) (Abcam, catalog number ab65359) and β-hydroxyburate (Colorimetric) (Abcam, catalog number ab83390), respectively, following manufacturer's instructions.

Optical coherence tomography (OCT) retinal imaging

To image cross-sections of the retina *in vivo*, mice were anesthetized and pupils were dilated with 1% tropicamide for 5 min and GenTeal eye gel was applied freely throughout the procedure to prevent corneal dryness. OCT images were acquired using a Heidelberg Spectralis HRA + OCT system (Heidelberg Engineering, Heidelberg, Germany) (In vivo detecting mouse persistent hyperplastic primary vitreous by Spectralis Optical Coherence Tomography - PubMed (nih.gov)).

In vivo retinal imaging using MICRON III

To image the fundus, mice were anesthetized and eyes were dilated with 1% tropicamide for 5 min and kept hydrated with GenTeal, as described above. Fluorescence was imaged on a Micron III

retinal imaging microscope (Phoenix Research Labs, Pleasanton, CA) using an ET620/60X emission filter (Chroma Technology Corp., Bellows Falls, VT). Using ImageJ, the average intensity of the fluorescence on the back of the retina was quantified.

Electroretinogram – photopic

For photopic, light-adapted, ERGs, the procedure was identical to the one described in the main text for **Electroretinography**, except that each eye of each mouse was separately exposed to 15 flashes of 10 Hz, 1 candela-seconds per meter squared (cd.s/m2).

Figure S1.

A.

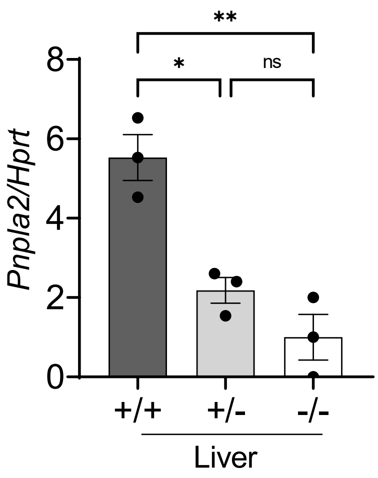

B.

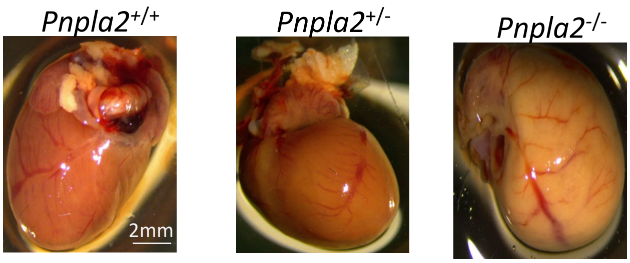

C.

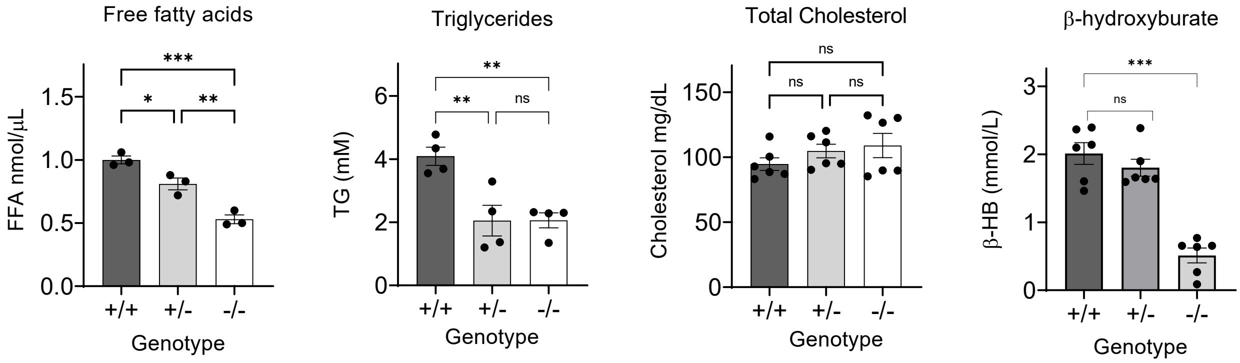

Anatomical assessment of heart and plasma parameters

A. *Pnpla2* expression (relative to *Hprt*) in liver, from 3 months old *Pnpla2*$^{+/+}$, *Pnpla2*$^{+/-}$ and *Pnpla2*$^{-/-}$ mice. B. Photographs of hearts from 3-months old *Pnpla2*$^{+/+}$, *Pnpla2*$^{+/-}$ and *Pnpla2*$^{-/-}$ mice. The white discoloration is due to lipid accumulation in the heart of *Pnpla2*$^{-/-}$ mice. C. Free fatty acids (FAA), triglycerides (TG), cholesterol and β-hydroxybutyrate levels were determined in plasma of 12-week-old from *Pnpla2*$^{+/+}$, *Pnpla2*$^{+/-}$ and *Pnpla2*$^{-/-}$. Each data point corresponds to values from one animal. For all assays shown, n = 3-6 retinas per group. *P < .05, **P < .001, ***P < .0001, ns P> 0.05

Figure S2.

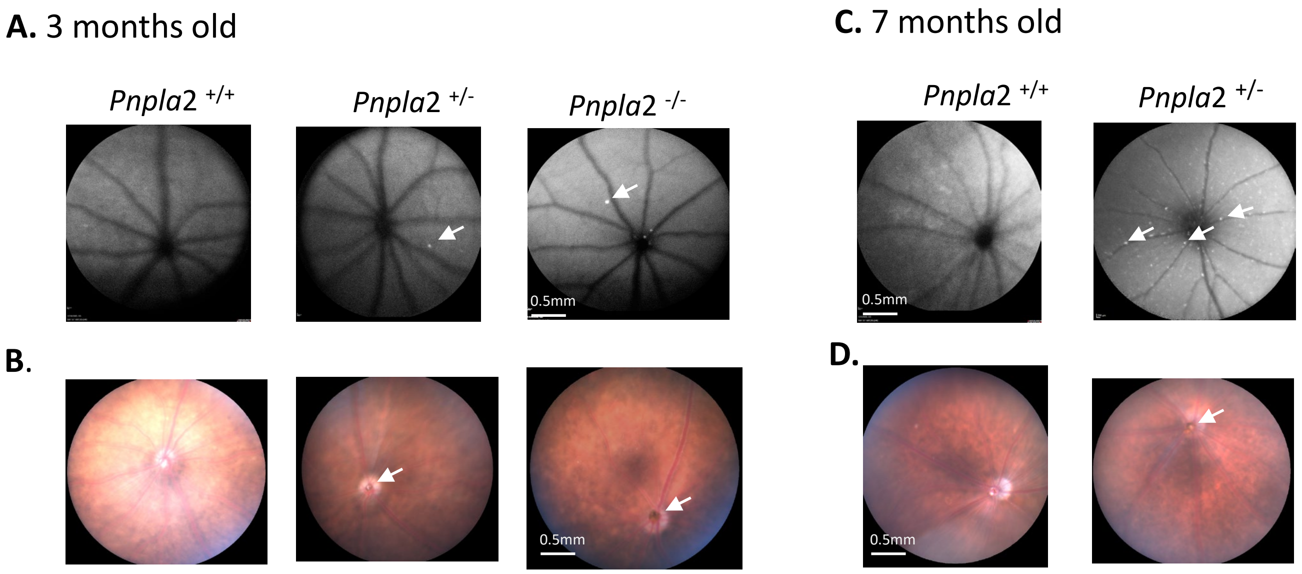

Visual assessment on *Pnpla2*-KO mouse line
A. and C. Angiography micrographs of retinas of *Pnpla2*$^{+/+}$, *Pnpla2*$^{+/-}$ and *Pnpla2*$^{-/-}$ mice at 3- and 7-months of age. *Pnpla2*$^{+/-}$ at 7 months of age showed the presence of multiple white lesions located at the deeper levels of the retina (white arrows).
B. and D. Fundoscopy micrographs of retinas of *Pnpla2*$^{+/+}$, *Pnpla2*$^{+/-}$ and *Pnpla2*$^{-/-}$ mice at 3- and 7-months of age. *Pnpla2*$^{+/-}$ at 3- and 7-months of age and *Pnpla2*$^{-/-}$ at 3-months of age show the presence of an optic nerve disc pit defect next to the optic nerve. For all experiments shown, n = 3 per group.
Scale bar corresponds to 0.5 mm with respect to size of a mouse eye.

Figure S3.

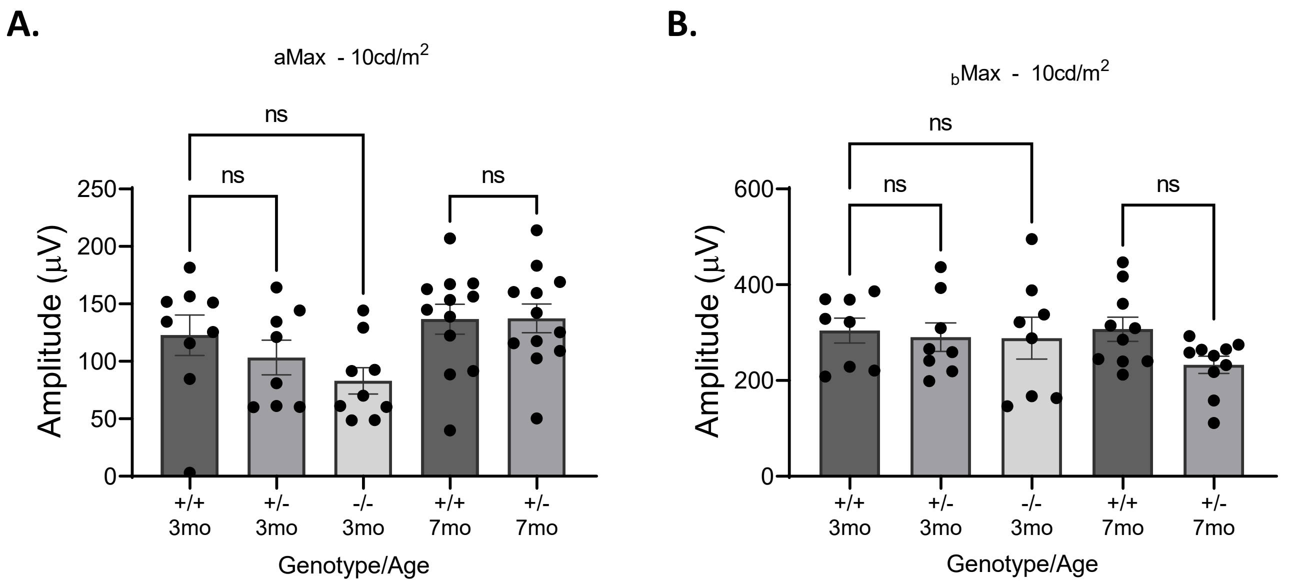

PEDF-R deficiency did not alter photopic retinal function.
A. and B. Graphs showing photoreceptor a-wave amplitudes and b-wave amplitudes, respectively, at 10cd.s/m^2 of each genotype and age of mice. For all ERG data shown, n = 8 for *Pnpla2*$^{+/+}$, n = 8 for *Pnpla2*$^{+/-}$ and n = 9 for *Pnpla2*$^{-/-}$. For groups at 7 months of age, all ERG data show n = 10 for *Pnpla2*$^{+/+}$ and n = 10 for *Pnpla2*$^{+/-}$. Each data point corresponds to the amplitude of one mouse. $*p < 0.05$, $**p < 0.001$, $****p < 0.00001$.
